# H19 inhibition increases HDAC6 and regulates IRS1 levels and insulin signaling in the skeletal muscle during diabetes

**DOI:** 10.1186/s10020-022-00507-3

**Published:** 2022-07-16

**Authors:** Amit Kumar, Malabika Datta

**Affiliations:** 1 CSIR-Institute of Genomics and Integrative Biology, Mall Road, Delhi, 110007 India; 2 Academy of Scientific and Innovative Research, CSIR-HRDC, Kamala Nehru Nagar, Ghaziabad, 201002 Uttar Pradesh India

**Keywords:** Epigenetics, lncRNA H19, HDAC6, Skeletal muscle, Insulin resistance, Diabetes, IRS1

## Abstract

**Background:**

Histone deacetylases (HDACs) that catalyze removal of acetyl groups from histone proteins, are strongly associated with several diseases including diabetes, yet the precise regulatory events that control the levels and activity of the HDACs are not yet well elucidated.

**Methods:**

Levels of H19 and HDACs were evaluated in skeletal muscles of normal and diabetic db/db mice by Western Blot analysis. C2C12 cells were differentiated and transfected with either the scramble or H19 siRNA and the levels of HDACs and *Prkab2*, *Pfkfb3*, *Srebf1*, *Socs2*, *Irs1* and *Ppp2r5b* were assessed by Western Blot analysis and qRT-PCR, respectively. Levels of H9, HDAC6 and IRS1 were evaluated in skeletal muscles of scramble/ H19 siRNA injected mice and chow/HFD-fed mice.

**Results:**

Our data show that the lncRNA H19 and HDAC6 exhibit inverse patterns of expression in the skeletal muscle of diabetic db/db mice and in C2C12 cells, H19 inhibition led to significant increase in HDAC activity and in the levels of HDAC6, both at the transcript and protein levels. This was associated with downregulation of IRS1 levels that were prevented in the presence of the HDAC inhibitor, SAHA, and HDAC6 siRNA suggesting the lncRNA H19-HDAC6 axis possibly regulates cellular IRS1 levels. Such patterns of H19, HDAC6 and IRS1 expression were also validated and confirmed in high fat diet-fed mice where as compared to normal chow-fed mice, H19 levels were significantly inhibited in the skeletal muscle of these mice and this was accompanied with elevated HDAC6 levels and decreased IRS1 levels. In-vivo inhibition of H19 led to significant increase in HDAC6 levels and this was associated with a decrease in IRS1 levels in the skeletal muscle.

**Conclusions:**

Our results suggest a critical role for the lncRNA H19-HDAC6 axis in regulating IRS1 levels in the skeletal muscle during diabetes and therefore restoring normal H19 levels might hold a therapeutic potential for the management of aberrant skeletal muscle physiology during insulin resistance and type 2 diabetes.

## Introduction

Glucose homeostasis is a crucial contributor towards the survival of an organism and therefore impairment or prolonged disturbance of this key process results in serious metabolic aberrations. One of the major tissues involved in utilizing glucose and maintaining its levels is the skeletal muscle where almost 80% of post-prandial glucose disposal takes place and it is considered as the prime site for insulin dependent glucose uptake (DeFronzo et al. 1981). Insulin resistance due to impaired insulin signaling interferes with such insulin stimulated glucose uptake and the skeletal muscle highly ranks among the significant contributory primary sites for such an impairment that results in decreased glucose uptake and consequently manifests in the onset and progression of insulin resistance and diabetes. Ninety percent of all diabetic cases are of type II, potentially because of significant environmental influence with epigenetic events being at the center of the several underlying causes. Among the epigenetic events, histone acetylation and DNA/histone methylation are the most aggressively studied modifications in terms of their roles in normal and diseased states. In the last few years, dynamics of chromatin accessibility by virtue of histone acetylation/deacetylation has been greatly studied in almost all aspects of diabetes (Gray and Meyts 2005; Li et al. 2016; Zhong and Kowluru 2010). The reversible affair of histone acetylation and deacetylation balanced by histone acetylases (HATs) and histone deacetylases (HDACs), respectively, strongly influences gene expression, cell fate and survival and therefore, an imbalance in the expression of these critical enzymes exerts serious deleterious consequences (Timmermann et al. 2001). Diabetic complications are associated with elevated expression of HDACs (Christensen et al. 2011; Dewanjee et al. 2021) that are strong protagonists in skeletal muscle physiology and insulin resistance (Tian et al. 2020). Downregulation of HDACs has been shown to assist in skeletal muscle insulin sensitivity and pharmacological inhibition of HDACs has been postulated as a viable therapeutic intervention for diabetes (Christensen et al. 2011; Dewanjee et al. 2021).

Recently, while non-coding RNAs (ncRNAs), in general, have been identified as strong players and regulators in epigenetic events (Wei et al. 2017), the regulatory functions of long non-coding RNAs (lncNRAs) are still not completely understood. Although few reports demonstrate regulation of the epigenetic machinery by lncRNAs, the diverse mechanisms that lncRNAs employ in cell function calls for detailed and specific investigations. The lncRNA, XIST is required for X-chromosome inactivation, which is one of the most classical epigenetically-regulated phenomena (Froberg et al. 2013). Antisense to the lncRNA XIST to its 5′ end, lies the lncRNA, TSIX that interacts with the PRC2 complex and aids hypermethylation of DNA through DNMT3A, thereby silencing lncRNA XIST and preventing X-chromosome inactivation on the other X-chromosome of the pair (Froberg et al. 2013). LncRNAs MALAT1 and FENDRR epigenetically repress gene expression by binding to the histone methyltransferase complex, PRC2 and consequently promote hypermethylation of H3K27 on the target gene promoter (Said et al. 2021; Grote and Herrmann 2013). Similarly, oncogenic lnRNAs namely HULC, UCA1 and PVT1 epigenetically silence the expression of certain genes such as NKD2, p21, E-cadherin, p15, p16 via H3K27 trimethylation by directly binding to EZH2 of the PRC2 complex in colorectal carcinoma, gallbladder and gastric cancer (Cai et al. 2017; Kong et al. 2015; Yang et al. 2016). Chromatin dynamics by dint of histone methylation is also regulated by the lncRNA, HOTAIR that directly interacts with key epigenetic regulators, the PRC2 complex and histone demethylase LSD1, to regulate gene silencing (Bhan and Mandal 2015). Similarly, lncRNAs DACOR1, ANRIL, MEG3, GAS5, NEAT1, TP53TG1, H19 and GCLnc1 have also been shown to epigenetically modify and regulate gene expression in various cancer types (Ayub and D’Angelo Papaiz 2019). The maternally expressed lncRNA, H19 has been extensively studied in the skeletal muscle and its role in the skeletal muscle during diabetes has been identified (Dey et al. 2014; Gao et al. 2014; Geng et al. 2018; Gui et al. 2020; Zhang et al. 2020a). Decreased levels of the lncRNA, H19 have been reported in the skeletal muscle of diabetic human subjects and HFD fed mice and this negatively influences the insulin signaling cascade and glucose uptake (Gao et al. 2014), suggesting that the decreased levels of H19 impair skeletal muscle insulin signaling and thereby contribute to aberrant glucose metabolism in this tissue during diabetes.

In this study, we sought to explore if decreased levels of H19 might regulate histone modifications and impair metabolic pathways in the skeletal muscle during diabetes. We show that H19 regulates the levels of HDAC6 within the cell and this regulation consequently determines IRS1 levels and insulin resistance in the skeletal muscle.

## Materials and methods

### Animal experiments

Ten-twelve week-old male normal (C57BLKs− db/ + ; weight: 23.5 ± 1.41 g; blood glucose levels: 119 ± 28.5 mg/dl) and diabetic (C57BLKs− db/db; weight: 35.70 ± 6.0 g; blood glucose levels: 354.25 ± 55.5 mg/dl) mice (n = 6) were obtained from the CSIR-Central Drug Research Institute (CSIR-CDRI), Lucknow, India. Mice were kept in temperature controlled, pathogen free barrier facility at a 12:12 h light–dark cycle at the CSIR-Institute of Genomics and Integrative Biology (CSIR-IGIB), New Delhi (India) and were given ad-libitum access to diet and water. Mice were sacrificed using thiopentone and skeletal muscle tissues from both groups of mice were rapidly isolated and stored in RNA later (Ambion, Life Technologies, CA, USA) or frozen at − 80 °C until further use. To study the effect of H19 in-vivo, skeletal muscle tissues from either scramble or H19 siRNA injected mice, as described by Goyal et al. were taken (Goyal et al. 2019). For generating high fat diet fed (HFD) mice model, seven week-old male mice (C57BL/6,n = 6) were obtained from the animal house facility, CSIR-IGIB, New Delhi and were maintained in a temperature controlled, pathogen free barrier facility with access to food and water at a 12:12 h light–dark cycle. Animals were randomly divided in two groups and fed either a high fat diet (HFD; 60% kcal of fat; Research diet, Inc, USA) or normal chow diet (10% kcal of fat) for a period of 6 months. Body weight and glucose levels were significantly higher in the HFD group (body weight: 52.16 ± 3.46 g; blood glucose: 219.6 ± 34.12 mg/dl) as compared to normal chow diet fed mice (body weight: 30.2 ± 1.52 g; blood glucose: 160 ± 18.93 mg/dl). After 6 months, mice were sacrificed using thiopentone and skeletal muscle tissues from both normal and HFD mice were isolated and stored in RNA later (Ambion, Life Technologies, CA, USA) at − 80 °C until further use. All experimental procedures were performed according to the guidelines of the Committee for the Purpose of Control and Supervision of Experiments on Animals (CPCSEA), New Delhi, India and were approved by Institutional Animal Ethics Committee (IAEC) of CSIR-IGIB, New Delhi, India.

### Cell culture and transfections

C2C12 mouse skeletal muscle cells were obtained from the National Centre for Cell Science (NCCS), Pune, India and maintained in Dulbecco’s modified Eagle’s medium (DMEM) containing high glucose (Sigma, St. Louis, USA), supplemented with 10% Fetal Bovine Serum (Gibco, USA) and 10 units/ml streptomycin/penicillin/glutamine (Gibco, USA). At a confluence of approximately 70–80%, the media was replaced with differentiation media (DMEM-high glucose with 2% horse serum, Gibco, USA) that facilitated the conversion of C2C12 myoblasts into elongated and tubular myotubes by 4–5 days. Endogenous levels of the lncRNA H19 was knocked down by transfection with H19 siRNA (1–5 nM, GE Dharmacon, USA) and control cells were transfected at equi-concentration with scramble (Scr) using Lipofectamine RNAiMax (Invitrogen, CA, USA). After an overnight incubation, the culture medium was replaced with fresh DMEM and 48 h later, total RNA and protein was isolated for further experiments as described below. To study if effects of H19 are mediated through HDACs, C2C12 cells transfected with either the scramble or H19 siRNA were incubated for 24 h in the absence or presence of SAHA (10 µM), a well-known HDAC inhibitor. To evaluate the specific mediatory role of HDAC6 in the effects of H19 inhibition on IRS1 levels, C2C12 cells were co-transfected with the HDAC6 siRNA (25 nM, SantaCruz, TX, USA) and H19 siRNA using Lipofectamine RNAiMax (Invitrogen, CA, USA). After 48 h, total RNA and protein was isolated for qRT-PCR and Western Blot as described below.

### Bioinformatic analyses

TargetScan (http://www.targetscan.org) and miRDB (http://mirdb.org/) were used as microRNA target prediction tools to predict genes targeted by miR-675-5p and miR-675-3p. The presence of histone marks on gene promoters were analyzed through the UCSC genome browser (https://genome.ucsc.edu/). H19-HDAC6 RNA-RNA interactions were analyzed using the LncRRIsearch web server (http://rtools.cbrc.jp/LncRRIsearch/).

### HDAC activity assay

HDAC activity in scramble and H19 transfected tissues or cells was estimated using the HDAC activity kit (BioVision, Inc., CA, USA) according to the manufacturer’s protocol. Briefly, 90 µg of tissue or cell lysates (lysed in RIPA lysis buffer with protease inhibitor) along with deacetylated standard (provided with the kit) was incubated in the presence of assay buffer and substrate. All samples were incubated for 1 h at 37 °C and then read at 400 nm in a plate reader (Tecan, Mannedorf, Switzerland). Total protein was estimated using BCA protein estimation kit (GBiosciences, USA). HDAC activity is expressed as µM of substrate deacetylated per µg of protein.

### RNA isolation and qRT-PCR

Total RNA was isolated from mice skeletal muscle tissues and from cells transfected with either the scramble or H19 siRNA, using TriZol (Invitrogen, CA, USA). cDNA was synthesized using RevertAid reverse transcriptase (Thermofisher Scientific, USA) using 1 µg RNA (cells or tissues) and transcript levels of genes, *Prkab2, Pfkfb3, Srebpf1, Socs2, Irs1, Ppp2r5b* along with *Hdac1-6* and *lncRNA H19* were quantified by qRT-PCR using specific primers (Table 1) and PowerUp SYBR Green Master mix (Applied Biosystems, Life Technologies, USA) in an Applied Biosystems Step One Plus and/or QuantStudio 6 Real Time PCR (Applied Biosystems, Life Technologies, USA). 18S rRNA was used as a normalization control and reaction specificity was determined by melt curve analysis. cDNA to quantify miR-675-5p and miR-675-3p levels were prepared using specific stem-loop RT primers (Table 1) and PCR was performed as described above with miRNA specific primers. Sno-234 was used as the normalization control. Transcript levels were analyzed with threshold cycle (Ct) values using 2^−∆∆Ct^ method.

**Table 1:** Primer sequences used for qRT-PCR and cloning

Gene name	Primer sequence [5′–3′]
H19	Forward: CCTCAAGATGAAAGAAATGGTGCTA
	Reverse: TCAGAACGAGACGGACTTAAAGAA
Hdac1	Forward: GACGGCATTGACGACGAATC
	Reverse: TGAAGCAACCTAACCGGTCC
Hdac2	Forward: GGAATGTTGCCCGATGTTGG
	Reverse: TGCAGTTTGAAGTCTGGTCC
Hdac3	Forward: TTCCCTCAAACTCTCACGGC
	Reverse: AGTTGCTGGGGCTCATTACC
Hdac4	Forward: CCCGCTACTGGTATGGGAAG
	Reverse: GGTGGTTGTAGGAGGCTGAC
Hdac5	Forward: GAGGGAGGCCATGACTTGAC
	Reverse: TCTCTAGTGTGGCAACCGC
Hdac6	Forward: ACTGCACTGCCCATGGATAC
	Reverse: GTAGGATGAGACAGCGAGCC
Prkab2	Forward: TCACCTACCTCCTGGCTCTC
	Reverse: AGAGTCTCAGCTGAGGGAGG
Pfkfb3	Forward: GTCGCCGAATACAGCTACGA
	Reverse: GAGCCCCACCATCACAATCA
Socs2	Forward: CAGCTGGACCGACTAACCTG
	Reverse: GGTGAACAGTCCCATTCCGT
Srebf1	Forward: TAGTGACTCTGAGCCCGACA
	Reverse: ATCTCTGCTCTCTGCCTCCA
Irs1	Forward: TGGACATCACAGCAGAATGAAGA
	Reverse: GACGTGAGGTCCTGGTTGTG
Ppp2r5b	Forward: TTGACCCGTGTTGTCCACTG
	Reverse: AGGCTGGCACATCTTTGAGC
18S rRNA	Forward: AGAAACGGCTACCACATCCA
	Reverse: CCCTCCAATGGATCCTCGTT
miR-675-5p StemLoop	CTCAACTGGTGTCGTGGAGTCGGCAATTCAGTTGAGACTGTGGG
miR-675-5p FP	Forward: ACACTCCAGCTGGGTGGTGCGGAAAGGGCC
miR-675-3p StemLoop	CTCAACTGGTGTCGTGGAGTCGGCAATTCAGTTGAGACTGAGCG
miR-675-3p FP	Forward: ACACTCCAGCTGGGCTGTATGCCCTAACC
Universal RP for miR-675-5p/3p	Reverse: CTCAATCGTACATAGAAACAGGGATC
Sno-234 StemLoop	GGATCGCCTCTCAGTGGTAG
Sno-234	Forward: GGCTTTTGGAACTGAATCTAAGT
	Reverse: GAGGTATTCGCACCAGAGGA
HDAC6 Cloning primers	Forward Kpn1 site: CGG**GGTACC**ATGACCTCCACCGGCCAAGATTC
	Reverse EcoR1 site: CCG**GAATTC**GTGTGAGTGGGGCATGTCCTC

*FP* forward primer, *RP* reverse primer

Inserted restriction enzyme sites are indicated in bold and underlined letters in the HDAC6 cloning primers

### Western blotting

Scramble or H19 siRNA transfected C2C12 cells were lysed in RIPA Buffer (Sigma, St. Louis, USA) containing protease inhibitors (Calbiochem, Darmstadt, Germany) and total protein content was measured by the BCA protein assay kit (786–571; G-Biosciences, USA. Protein samples (20-30 µg) were resolved on SDS-PAGE and analyzed by Western Blotting using primary antibodies against HDAC1 (10E2; Cell Signaling Technology, USA), HDAC2 (3F3; Cell Signaling Technology, USA), HDAC3 (7G6C5; Cell Signaling Technology, USA), HDAC4 (D15C3; Cell Signaling Technology, USA), HDAC5 (2082P; Cell Signaling Technology, USA), HDAC6 (7612S; Cell Signaling Technology, USA), p-HDAC6-Ser22 (PA5-38498; Invitrogen, USA), IRS1 (2390; Cell Signaling Technology, USA). HSC70 (7298; Santa Cruz Biotechonology,Inc, USA) was used as a loading control. Appropriate HRP conjugated anti-rabbit (105499; GeNei Laboratories, India) and anti-mouse (62114058001A; GeNei Laboratories, India) secondary antibodies were used to detect the immunoreactive bands using the ECL western blotting kit (Pierce, Thermo Scientific, Rockford, IL, USA). AlphaEaseFC software (Alpha Innotech Corporation, USA) was used for densitometric analysis and the pixel density of each band was normalized to the background intensity.

To evaluate the effects of H19 on insulin signaling, differentiated C2C12 cells were transfected with either the scramble or H19 siRNA (5 nM). Cells were serum starved for 12 h and incubated in the presence or absence of insulin (100 nM; 20 min). On termination of the incubation, cells were lysed in RIPA Buffer containing phosphatase and protease inhibitors (Calbiochem, Darmstadt, Germany) and 20–30 µg protein was subjected to western blotting to evaluate the levels of IRS1 (2390S; Cell Signaling Technology, USA), p-IRS1-Tyr612 (ab66153; Abcam, USA), Akt (4685S; Cell Signaling Technology, USA) and p-Akt-Ser473 (4060S; Cell Signaling Technology, USA) as described above. Vinculin (73614; Santa Cruz Biotechonology,Inc, USA) was used as the normalizing control.

### Full length cloning of HDAC6

Full length coding sequence of mouse HDAC6 was cloned into pcDNA3.1 + overexpression vector (Thermo Fisher Scientific, USA) using specific primers (Table 1). Differentiated C2C12 cells were transfected with either the control vector or the HDAC6 overexpression vector using Lipofectamine 2000 (Invitrogen, USA) as per the manufacturer’s protocol. After 48 h, cells were lysed and total RNA and protein was isolated for assessing levels of HDAC6 and IRS1 by qRT-PCR and Western Blot analysis.

### Statistical analysis

Student’s t-test was used to calculate the statistical significance and the values were represented as means ± standard deviation (SD). P-value ≤ 0.05 was considered as statistically significant.

## Results

### LncRNA H19 levels are down-regulated and HDAC levels are up-regulated in the diabetic mice skeletal muscle

**Fig. 1:**
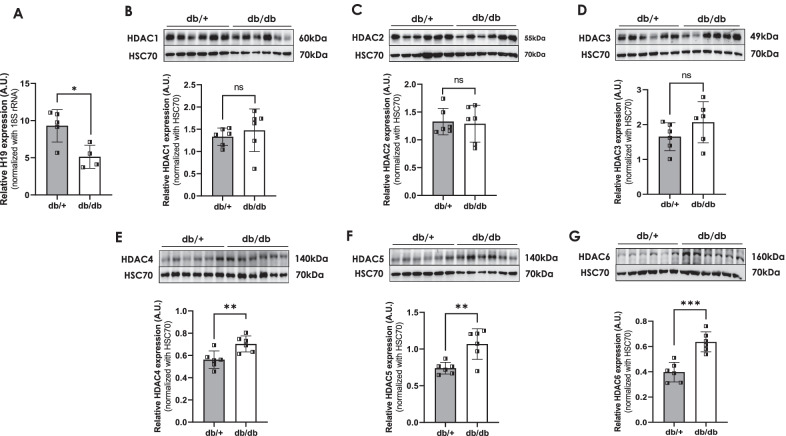
Expression of lncRNA H19 and HDACs in db/db mice skeletal muscle. **A** Total RNA was isolated from the skeletal muscle of normal (db/ +) and diabetic (db/db) mice and 1 µg RNA was reverse transcribed and subjected to qRT-PCR to assess the transcript levels of the lncRNA H19. 18S rRNA was used as the loading control. Skeletal muscle of normal (db/ +) and diabetic (db/db) mice were lysed as described in the “Material and methods” section and 20–30 µg lysates were run on SDS-PAGE and the levels of HDAC 1–6 were evaluated by Western Blot analysis. HSC70 was used as the loading control. Densitometric analyses of the expression are shown in the panel below (**B**–**G**). All experiments were performed in at least four animals in each group, values are means ± SD. *p ≤ 0.05; **p ≤ 0.01; ***p ≤ 0.001

In this study, we sought to explore the epigenetic effects of lncRNA H19 in the skeletal muscle during diabetes. LncRNA H19 levels are significantly downregulated in the skeletal muscles of diabetic db/db mice (Fig. 1A). Similar patterns of lncRNA H19 inhibition in the skeletal muscle of diet induced HFD mice and diabetic human subjects have been reported by Gao et al. (2014). Epigenetic histone modifications are considered as major underlying mechanisms for sustained metabolic aberrations in diabetes and HDAC inhibition is viewed as a novel treatment strategy for diabetes (Christensen et al. 2011; Dewanjee et al. 2021). Here we demonstrate that while protein levels of HDAC 4, 5 and 6 are significantly up-regulated in the skeletal muscle of diabetic mice, the levels of HDAC 1, 2 and 3 are unchanged (Fig. 1B–G). Both lncRNA H19 and HDAC 4, 5 and 6 are crucial in skeletal muscle physiology (Zhang et al. 2020a; Ratti et al. 2015; Lu et al. 2000) and such inverse patterns of expression between the lncRNA, H19 and HDAC 4, 5 and 6 indicated towards a potential regulatory axis between them that might be correlated to aberrant skeletal muscle metabolism during diabetes.

### LncRNA H19 inhibition upregulates HDAC6 levels in mouse C2C12 cells

**Fig. 2:**
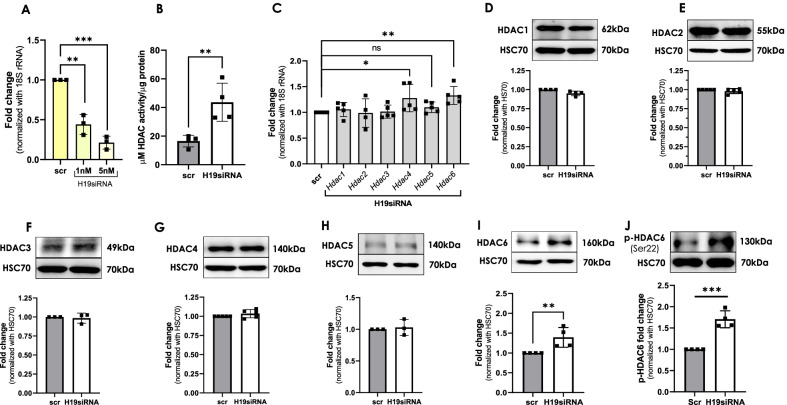
H19 inhibition regulates HDAC6 levels in C2C12 cells. **A** Differentiated C2C12 cells were transfected with either the scramble or H19 siRNA (1–5 nM) and after 48 h, total RNA was isolated and the transcript levels of H19 were assessed by qRT-PCR. 18S rRNA was used as the loading control. **B** Differentiated C2C12 cells were transfected with either the scramble or H19 siRNA as in “A” and after 48 h, cells were lysed and 90 µg cell lysates were used to assay HDAC activity as described in the “Material and methods” section. HDAC activity is expressed as µM of substrate deacetylated per µg of protein. **C** Total RNA was isolated from the C2C12 cells transfected as in “A” and the transcript levels of HDAC 1–6 were evaluated by qRT-PCR. 18S rRNA was used as the loading control. C2C12 cells transfected as in “A” were lysed and lysates (20–30 µg) were subjected to Western Blot analyses to evaluate the levels of HDAC 1–6. HSC70 was used as the loading control (**D**–**I**). **J** 20 µg of lysate from scramble and H19 siRNA transfected C2C12 cells was subjected to western blot analysis to probe the expression of pHDAC6-Ser22. HSC70 was used as the loading control. Figures presented are representative images and values are means ± SD of at least three independent experiments. *p ≤ 0.05; **p ≤ 0.01; ***p ≤ 0.001; ns: non-significant

Since, we observed an inverse pattern of expression between H19 and HDAC 4, 5 and 6 in the db/db mice skeletal muscle, we investigated if H19 might regulate HDAC levels. H19 levels were inhibited in C2C12 skeletal muscle cells using specific siRNA and the levels of HDACs were evaluated. C2C12 cells were transfected with H19 siRNA (1–5 nM) and this led to a dose dependent inhibition of endogenous H19 levels with around 80% inhibition at the dose of 5 nM (Fig. 2A). Interestingly, as compared to scramble transfected cells, HDAC activity was significantly increased in cells transfected with H19 siRNA (Fig. 2B), suggesting a possible correlation between H19 inhibition and HDAC activity. While following the levels of HDACs in the presence of H19 siRNA, there was no significant change in the transcript or protein levels of HDAC 1–5 (Fig. 2C–H). Although H19 inhibition did exert a modest increase in the transcript levels of HDAC 4, this was not evident at the protein level. Interestingly, however, as compared to scramble transfected cells, there was a significant increase in the levels of HDAC6, both at transcript and protein levels (Fig. 2C, I) in the presence of H19 siRNA. HDAC6 is specifically activated by phosphorylation at Ser-22 (Du et al. 2015), we therefore evaluated the activation status of the increased levels of HDAC6. As compared to scramble transfected cells, there was a significant increase in the levels of p-HDAC6 (Ser-22) in the presence of H19 siRNA (Fig. 2J). All these results suggest that H19 inhibition increases HDAC6 levels and its activation in skeletal muscle cells. Hence, increased HDAC activity and HDAC6 levels as is observed in the skeletal muscle of diabetic db/db mice (Fig. 1) might be mediated by inhibited H19 levels.

### H19 effect on HDAC6 is not dependent on miR-675-5p/3p

**Fig. 3:**
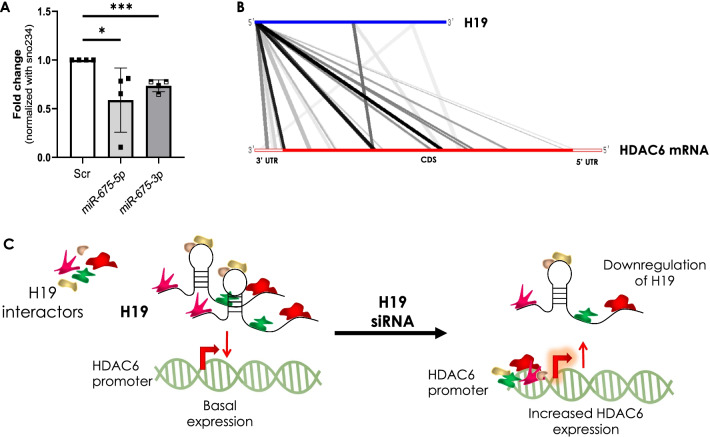
Schematic representation of potential regulatory mechanisms of HDAC6 by lncRNA H19. **A** Differentiated C2C12 cells were transfected with either the scramble or H19 siRNA (5 nM) and after 48 h, total RNA was isolated and the levels of miR-675-5p and miR-675-3p were assessed by qRT-PCR. Sno-234 was used as the loading control. Data are means of at least three independent experiments and values are means ± SD. *p ≤ 0.05, ***p ≤ 0.001. **B** Potential binding between the lncRNA H19 and HDAC6 mRNA as derived from the online LncRRIsearch prediction tool. UTR: Untranslated Region; CDS: Coding DNA sequence. **C** Binding of H19 to its interactors might prevent their occupancy on the HDAC6 promoter, thereby preventing its transcription. In the absence or decreased levels of H19, the interactors get free to bind to their potential binding elements on the HDAC6 promoter to increase HDAC6 gene transcription

We then sought to explore the possible mechanisms of HDAC6 regulation by H19. H19 encodes only the primary miRNA precursor for miR-675-5p and miR-675-3p (Dey et al. 2014) and therefore several of the cellular effects of H19 are mediated through miR-675 (Dey et al. 2014; Keniry et al. 2012; Li et al. 2015; Luo et al. 2019). We, therefore, explored if the effect of H19 on HDAC6 as described above might be mediated through miR-675-5p/3p. As compared to scramble transfected cells, there was a significant inhibition of miR-675-5p and miR-675-3p levels in the H19 siRNA transfected C2C12 cells (Fig. 3A), suggesting that H19 might exert its effect on HDAC6 through miR-675-5p/3p. We therefore, scanned miRNA target prediction tools, namely TargetScan and miRDB to search if HDAC6 is the target of miR-675-5p and miR-675-3p. However, none of these prediction tools reflected HDAC6 to be targeted by miR-675-5p or miR-675-3p. We therefore, contemplate that H19 inhibition increases HDAC6 levels independent of miR-675-5p or miR-675-3p. Since lncRNAs can also directly interact with RNAs, we employed “LncRRIsearch", a web server for comprehensive prediction of lncRNA-mRNA interactions (Fukunaga et al. 2019) to explore potential interactions between H19 and HDAC6 mRNA. As shown in Fig. 3B, a total of 39 base pairing interactions are predicted between H19 and the 5’UTR, coding DNA sequence and the 3′UTR of HDAC6 mRNA. Also, several H19 interactors might act as putative transcription factors of HDAC6 with specific binding signatures on the HDAC6 promoter. These suggest that possibly, in the presence of H19 within the cell, these interactors remain bound to H19 and therefore remain unavailable to bind to the promoter of HDAC6 and initiate transcription. However, in the presence of H19 siRNA when its levels within the cell are decreased, these interactors are free and they might bind to the HDAC6 promoter and initiate its transcription, thereby increasing its levels within the cell (Fig. 3C). Although these interactions require validation and in-depth investigation, they at least suggest towards a possible regulatory axis between H19 and HDAC6.

### H19 inhibition down-regulates IRS1 levels and impairs insulin signaling in C2C12 cells

**Fig. 4:**
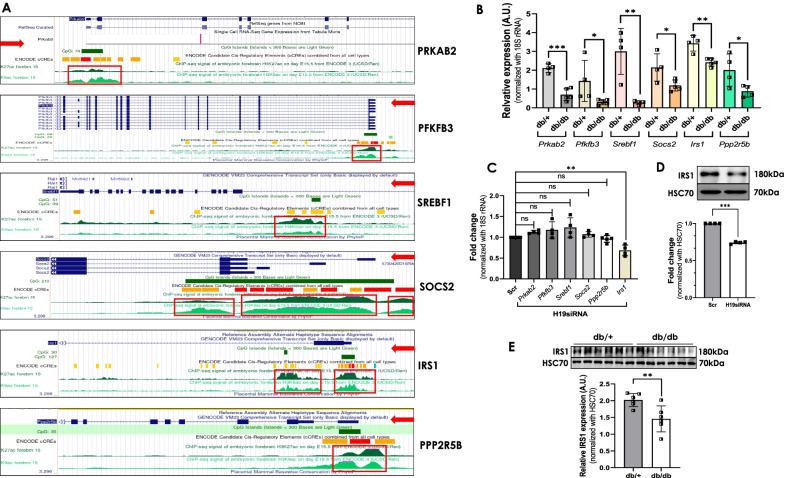
LncRNA H19 inhibition decreases IRS1 levels in C2C12 cells. **A** Putative acetylation marks as identified on the regulatory promoter regions of PRKAB2, PFKFB3, SREBF1, SOCS2, IRS1 and PPP2R5B using the UCSC genome database. Regions enclosed within the red box upstream to each gene represent the site with potential histone acetylation (K9 and K27) marks, red arrows represent the orientation of the gene. **B** Total RNA was isolated from skeletal muscle tissues of normal db/ + and diabetic db/db mice (n = 4) and 1 µg RNA was reverse transcribed and transcript levels of *Prkab2, Pfkfb3, Srebf1, Socs2, Irs1 and Ppp2r5b* were evaluated by qRT-PCR. 18S rRNA was taken as the normalization control. **C** C2C12 cells were differentiated and transfected with either the scramble or H19 siRNA (5 nM) and after 48 h, total RNA was isolated, reverse transcribed and the transcript levels of *Prkab2*, *Pfkfb3*, *Srebf1*, *Socs2*, *Irs1* and *Ppp2r5b* were assessed using gene specific primers. 18S rRNA was taken as the endogenous control. Experiments were done in at least four independent sets. **D** Cells transfected as in “C” were lysed and 20–30 µg protein was run on SDS-PAGE and subjected to Western Blot analysis using anti-IRS1 antibody. HSC70 was taken as the loading control. Experiments were done in four independent sets. **E** Skeletal muscle tissue lysates (30 µg) of normal db/ + and diabetic db/db mice (n = 6) were subjected to Western Blot analysis using anti-IRS1 antibody. HSC70 was taken as the loading control. Given below is the densitometric analysis of the normalized values of six mice in each group. Values presented are means ± SD of four independent experiments. *p ≤ 0.05; **p ≤ 0.01; ***p ≤ 0.001¸ns: non-significant

**Fig. 5:**
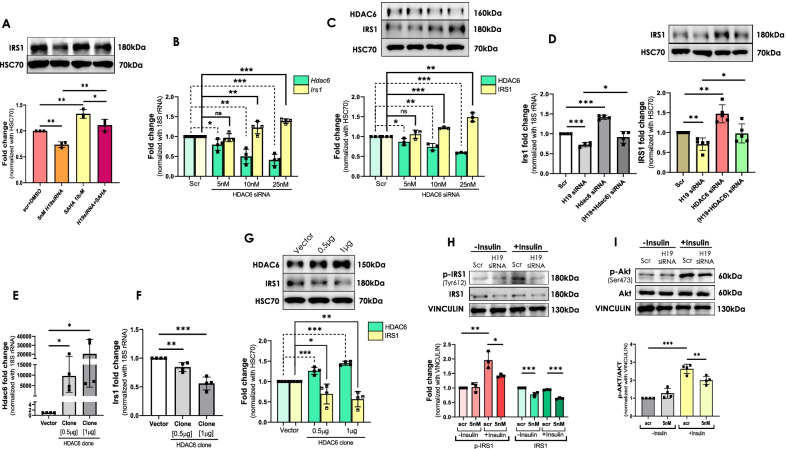
H19 inhibition regulates IRS1 levels in HDAC6 dependent manner and impairs insulin signaling in C2C12 cells. **A** Differentiated C2C12 cells were transfected with the scramble or H19 siRNA (5 nM) and treated with wither DMSO or the HDAC inhibitor, SAHA (10 µM). On termination of incubation (24 h), cells were lysed and 30 µg protein was resolved on SDS-PAGE and subjected to Western Blot analysis using anti-IRS1 antibody. HSC70 was used as the loading control. The blot presented is a representative blot and given below is the densitometric analysis of three independent experiments. **B** Differentiated C2C12 cells were transfected with either scramble or HDAC6 siRNA (5–25 nM) and after 48 h of transfection, cells were lysed and 1 μg of total RNA was reverse transcribed and the transcript levels of *Hdac6* and *Irs1* were detected by qRT-PCR. 18S rRNA was taken as the endogenous control. **C** Differentiated C2C12 cells were transfected as in “B” and after 48 h of transfection, 20 µg of protein was resolved on SDS-PAGE and probed using anti-HDAC6 and anti-IRS1 antibodies. HSC70 was used as the loading control. The blot presented is a representative figure and given below is the densitometric analysis of three independent experiments. **D** Differentiated C2C12 cells were transfected with the scramble or H19 siRNA (5 nM) alone or together with HDAC6 siRNA (25 nM). After 48 h of transfection, cells were lysed and 1 μg of total RNA was reverse transcribed and the transcript levels of *Irs1* were determined by qRT-PCR. 18S rRNA was taken as the endogenous control. Also, after a similar transfection, 20 µg of protein from scramble or H19 siRNA alone or together with HDAC6 siRNA transfected cells was resolved on SDS-PAGE and probed using anti-IRS1 antibody. HSC70 was used as the loading control. The blot presented is a representative figure and given below is the densitometric analysis of at least five independent experiments. C2C12 cells were transfected with either control vector or HDAC6 cloned vector (0.5 µg and 1 μg) and 48 h post-transfection, cells were lysed and the transcript levels of *Hdac6* (**E**) or *Irs1* (**F**) were determined by qRT-PCR. 18S rRNA was used as the endogenous control. **G** Differentiated C2C12 cells were transfected with either control vector or HDAC6 overexpression vector (0.5 µg and 1 μg) and 48 h post-transfection, cells were lysed and 20 µg lysate was resolved on SDS-PAGE and probed using anti-HDAC6 and anti-IRS1 antibody. HSC70 was used as the loading control. The blot presented is a representative figure and given below is the densitometric analysis of four independent biological replicates. Differentiated C2C12 cells were transfected with either the scramble or H19 siRNAs and after 48 h of transfection, cells were treated with insulin (100 nM, 20 min). On termination of incubation, cells were lysed and probed for IRS1, pIRS1 (**H**) and Akt, pAkt (**I**) protein levels by Western Blot analysis using specific antibodies. Vinculin was taken as the loading control. All experiments were done at least thrice and densitometric analyses of the same are provided. Values are means ± SD. *p ≤ 0.05; **p ≤ 0.01; ***p ≤ 0.001

Results until now suggest that H19 inhibition elevates HDAC6 levels. We hypothesized that such elevated HDAC6 expression would deacetylate histones, thereby compacting the chromatin and making it less accessible to transcription factors and consequently inhibiting genes’ transcription. To assess the physiological relevance of this regulation, we screened for down-regulated genes in the skeletal muscle of db/db mice from a previous study from our laboratory (Kesharwani et al. 2021). RNA sequencing of skeletal muscles from normal (db/ +) and diabetic (db/db) mice identified 109 genes to be down-regulated during diabetes. Pathway analyses revealed AMPK and Insulin signaling pathways as most enriched pathways with a set of common genes namely, *Prkab2, Pfkfb3, Srebf1, Socs2, Ppp2r5b, Irs1* and *Ppp1r3a* (Kesharwani et al. 2021). Interestingly, all the genes except *Ppp1r3a*, were found to have potential histone acetylation marks on their promoters (Fig. 4A) suggesting towards a possibility of them being regulated by histone acetylation/deacetylation. The transcript levels of these genes in skeletal muscles of normal and diabetic mice were validated by qRT-PCR and as described previously (Kesharwani et al. 2021), the levels of these transcripts were found to be significantly down-regulated (Fig. 4B). We, therefore, evaluated the transcript levels of all these genes in the absence and presence of H19 siRNA. As shown in Fig. 4C, H19 inhibition, significantly decreased *Irs1* transcript levels while the transcript levels of *Prkab2, Pfkfb3, Srebf1, Socs2* and *Ppp2r5b* remained unchanged. This was also validated at the protein level and as in transcript level changes, IRS1 protein levels were significantly decreased in the presence of H19 siRNA as compared to that of scramble (Fig. 4D). Corroborating these, as compared to normal db/ + mice, protein levels of IRS1 were significantly down-regulated in the skeletal muscle of diabetic db/db mice (Fig. 4E). These results suggest that by regulating HDAC6 levels, H19 possibly decreases IRS1 levels that might affect skeletal muscle metabolism. To validate such mediatory role of HDAC6, we used SAHA, a HDAC inhibitor and HDAC6 siRNA to evaluate if H19 effects on IRS1 are attenuated. As shown in Fig. 5A, while H19 significantly decreased IRS1 protein levels, this decrease was significantly prevented in the presence of SAHA, suggesting that the observed H19 effects on IRS1 could be mediated by HDACs. We subsequently attempted to assess the role of HDAC6 specifically; C2C12 cells were transfected with varied doses of HDAC6 siRNA (5–25 nM) and in the presence of the siRNA, there was a dose dependent decrease in HDAC6 and this was accompanied by a dose dependent increase in IRS1, both at the transcript and protein levels (Fig. 5B–C). Interestingly, the inhibitory effect of H19 siRNA on IRS1 transcript and protein levels was significantly rescued in the presence of HDAC6 siRNA (Fig. 5D). To explore if HDAC6 overexpression alone is sufficient to affect IRS1 levels, we cloned the full length coding sequence of HDAC6 and as compared to the vector alone transfected cells, transfection with the HDAC6 clone significantly increased the endogenous levels of HDAC6 and this was associated with decreased IRS1, both at the transcript and protein levels (Fig. Fig. 5E–G). All these results suggest that the H19-HDAC6 axis is critical in determining cellular IRS1 levels. IRS1 is a 131 kDa protein and plays a key role in transmitting signals from insulin to intracellular PI3K/Akt and ERK MAP kinase pathways. Since H19 inhibition decreased IRS1 levels, we sought to explore its effects on insulin signaling. As shown in Fig. 5H,I, while insulin significantly increased pIRS1 and pAKT levels in scramble transfected cells, this effect was significantly abrogated in the presence of H19 siRNA, suggesting that decreased H19 levels, by inhibiting IRS1 levels, impairs insulin signaling in C2C12 cells.

### H19 inhibition increases HDAC6 levels and decreases IRS1 levels in the skeletal muscle in-vivo

**Fig. 6:**
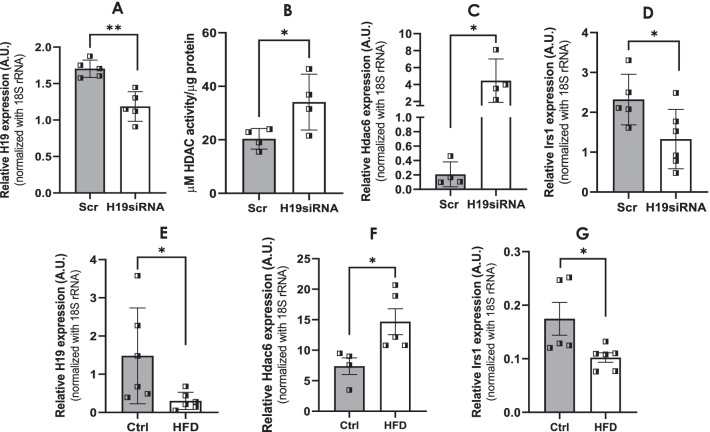
In-vivo H19 inhibition alters HDAC6 and IRS1 levels in the skeletal muscle of mice. **A** Mice were injected (i.v) with either the scramble or H19 siRNA (3 mg/kg body weight) as described in a previous study. Skeletal muscle tissues were isolated and the levels of H19 were analyzed by qRT-PCR. 18S rRNA was taken as the normalization control. **B** Skeletal muscle tissues from scramble and H19 siRNA injected mice were lysed and 90 µg of tissue was assayed for HDAC activity as described in the “Materials and methods” section. Values obtained were normalized to the total protein content. Total RNA (1 µg) isolated from skeletal muscle tissues of both groups of mice was reverse transcribed and the transcript levels of *Hdac6* (**C**) and *Irs1* (**D**) were assessed by qRT-PCR. 18S rRNA was taken as the loading control. Mice were fed either chow diet or high fat diet and after 6 months, skeletal muscle tissues were isolated total RNA extracted (1 µg), was reverse transcribed and the transcript levels of H19 (**E**), *Hdac6* (**F**) and *Irs1* (**G**) were evaluated by qRT-PCR. 18S rRNA was used as the loading control. Values presented are means ± SD of at least four animals in each group. *p ≤ 0.05; **p ≤ 0.01

A previous study from our laboratory had demonstrated that *in-vivo* administration of H19 siRNA induced hyperglycemia, hyperinsulinemia, hyperlipidemia and impaired oral glucose, insulin and pyruvate tolerance in mice (Goyal et al. 2019). Since, as described above H19 inhibition decreased IRS1 levels by increasing HDAC6 levels, we explored if this H19-HDAC6-IRS1 axis might be responsible for the observed metabolic anomalies as observed in H19 siRNA administered mice. As compared to scramble administered mice, H19 siRNA administration caused significant downregulation of H19 levels in the skeletal muscle (Fig. 6A). Together with this and consistent with the *in-vitro* results, there was a significant upregulation in HDAC activity in the skeletal muscle of H19 siRNA injected mice (Fig. 6B) and this was accompanied with elevated levels of *Hdac6* (Fig. 6C). Interestingly, as compared to scramble injected mice, there was a significant downregulation in the expression of *Irs1* in mice injected with H19 siRNA (Fig. 6D), thereby validating the data obtained in vitro and substantiating the significance of the H19-HDAC6-IRS1 axis as an important regulator in glucose homeostasis and metabolism. To confirm and validate these results in another model of diabetes, a high-fat diet (HFD) fed mice model was created by feeding a high fat diet (HFD) as described in the “Materials and Methods” section. As compared to chow diet fed mice, there was a significant downregulation of the lncRNA H19 in the skeletal muscle of HFD fed mice and this was associated with increased *Hdac6* levels and decreased *Irs1* levels (Fig. 6E–G). These data suggest a critical role of lncRNA H19 in the skeletal muscle during diabetes where, by altering HDAC6 levels, it modulates IRS1 levels and this might be one of the critical axes responsible for aberrant skeletal muscle physiology during diabetes.

## Discussion

Here, we present data to show that HDAC6 levels are regulated by the lncRNA H19 in the skeletal muscle during diabetes and this regulation modulates IRS1 levels, thereby exerting effects on insulin signaling.

HDACs are suggested as important regulators of physiological insulin signaling (Lee et al. 2020; Ye 2013). They catalyze the removal of acetyl groups from core histone proteins of nucleosomes and this makes the DNA template relatively inaccessible to transcription factors, thereby stalling transcription. Therefore, they are identified as vital regulators of diverse cellular events. HDAC levels and activity are precisely regulated at various levels and by multiple molecules, primarily through protein–protein interactions, post-transcriptional modifications, availability of cofactors, proteolytic cleavage etc. (Sengupta and Seto 2004).

During the past few years, non-coding RNAs (ncRNAs) have emerged as major regulators of gene expression, including those of HDACs. miR-206 regulates HDAC4 via transcriptional repression in amyotrophic lateral sclerosis during innervations (Williams et al. 2009). In an *in-vivo* model of Huntington’s disease, it was demonstrated that miR-22 targets HDAC4 and affects neuronal survival (Jovicic et al. 2013). Several microRNAs altered in Alzheimer’s disease target SIRT1, a class III HDAC and this affects learning and memory (Gao et al. 2010). HDAC1, being targeted by miR-449a, regulates cell growth and viability in prostate cancer cells (Noonan et al. 2009). The lncRNA, ANRIL, has been shown to act as a molecular scaffold between WDR5 and HDAC3, thereby regulating target gene expression (Zhang et al. 2020b).

In this study, we show that the levels of HDAC4-6 are increased in the skeletal muscle during diabetes and this is associated with decreased levels of lncRNA H19. Such inverse patterns of expression between the lncRNA and HDACs have been demonstrated in bone marrow mesenchymal stem cells (Huang et al. 2016). Mechanistically, however, our data show that H19 inhibition causes significant upregulation of only HDAC6, while the levels of HDAC4 and 5 remain unchanged. This indicates that elevated levels of HDAC6 as seen in skeletal muscle during diabetes are possibly mediated by decreased levels of lncRNA H19. H19 is a 2.3 kb paternally imprinted ncRNA, expressed from the maternal allele and is located in close proximity to the maternally imprinted, Igf2 locus (Gabory et al. 2010; Milligan et al. 2000). It is strongly expressed during embryogenesis but majorly repressed after birth, except in cardiac and skeletal muscles (Milligan et al. 2000). It induces transgenerational glucose intolerance in offsprings of gestational diabetic mice and impairs islet structure and function (Ding et al. 2012). Altered H19 levels in the skeletal muscle of human diabetic subjects and HFD mice impair insulin signaling and glucose uptake by acting as a molecular sponge of let-7 (Gao et al. 2014). In diet induced diabetic mice, hepatic H19 levels are chronically elevated and by regulating *Hnf4α* expression, they affect gluconeogenesis and hepatic glucose output (Zhang et al. 2018). This lncRNA prevents endothelial mesenchymal transition in diabetic retinopathy (Thomas et al. 2019) and by activating MPAK signaling, it accelerates wound healing in diabetic foot ulcers (Li et al. 2020). In a previous study from our laboratory, we had demonstrated that H19 levels are decreased in the livers of diabetic db/db mice and by regulating nuclear FOXO1 levels, they modulate hepatic gluconeogenesis and glucose output (Goyal et al. 2019).

The present study reveals an interesting aspect of HDAC6 regulation by the lncRNA H19 in the skeletal muscle during diabetes. However, the details of such a regulation are yet to be completely elucidated. This could be mediated through the involvement of miRNAs as demonstrated by Huang et al. (Huang et al. 2016), or the direct participation of H19 at transcriptional or translational levels. Towards examining the physiological relevance of this regulation, we scanned for histone acetylation marks on the promoters of downregulated genes in the skeletal muscle of db/db mice that were reported in a recent study from our laboratory (Kesharwani et al. 2021). Six genes, namely *Prkab2, Pfkfb3, Srebf1, Socs2, Ppp2r5b* and *Irs1* from the most enriched pathways (built from the set of genes downregulated in the skeletal muscle of db/db mice) were found to harbor acetylation marks on their promoters, suggesting that HDAC6 increase due to H19 inhibition in the db/db mice might deacetylate these genes’ promoters and consequently decrease their expression. H19 inhibition in C2C12 cells using specific siRNA significantly decreased IRS1 levels, while the expression of *Prkab2, Pfkfb3, Srebf1, Socs2* and *Ppp2r5b* were not altered, suggesting that H19-HDAC6 axis might be exerting its effect by decreasing IRS1 levels. Interestingly, the HDAC inhibitor, SAHA and specifically HDAC6 inhibition significantly rescued H19 mediated decrease in IRS1 levels, thereby potentiating the role of H19-HDAC6 in determining the cellular IRS1 levels. H19 inhibition significantly impaired insulin stimulated IRS1 and Akt phosphorylation, suggesting towards major blunting of insulin signaling in C2C12 cells in the presence of H19 siRNA. *In-vivo* inhibition of H19 significantly decreased skeletal muscle H19 levels along with increased HDAC6 and decreased IRS1 levels.

IRS1, encoded by the IRS1 gene is a 131 kDa protein and has a Pleckstrin homology (PH) domain at the N-terminus and phospho-tyrosine binding (PTB) at C-terminus. It plays a key role in the insulin signaling pathway and conveys signals from the insulin receptor via tyrosine phosphorylation to PI3K and AKT. Decreased IRS1 levels and consequently reduced tyrosine phosphorylation have been reported in 30% of the first-degree relatives (high risk individuals) of type II diabetic and obese subjects (Sesti et al. 2001; Smith 2002). IRS1 plays a major role in the insulin dependent glucose uptake in the skeletal muscle (Sesti et al. 2001). Various polymorphisms have been reported in the IRS gene family (namely, IRS1, IRS2, IRS3 and IRS4). Among all the polymorphisms studied within the IRS genes, Gly → Arg^972^ substitution in IRS1, along with environmental factors, appear to have a contributory role in the pathogenesis of type II diabetes (Sesti et al. 2001). Till now > 400 genetic variants have been identified for type II diabetes through genome wide association studies (Cai et al. 2020). Interestingly, one such genetic variant (the C allele of rs2943641) present in close proximity to IRS1 is associated with hyperinsulinemia and insulin resistance by negatively impacting the basal IRS1 levels and impairing insulin signaling in human skeletal muscle biopsy samples (Rung et al. 2009).

To conclude, our study identifies the lncRNA H19 as a critical determiner of cellular IRS1 levels and during diabetes, decreased levels of this lncRNA promotes an inhibition in IRS1 gene expression, possibly due to increased deacetylation mediated by HDAC6 in the skeletal muscle. Taken together, these events are critical in impaired skeletal muscle metabolism as observed in obesity and diabetes and emerge as major contributors of the abnormal physiology within this tissue.

## Conclusions

This study identifies the lncRNA H19-HDAC6 axis as a significant regulator of cellular IRS1 levels and impaired levels of lncRNA H19 and HDAC6 alter IRS1 levels in the skeletal muscle during diabetes. This consequently dampens insulin signaling and therefore targeting this interaction puts forth potential therapeutic strategies to normalize aberrant insulin signaling and metabolism within the skeletal muscle as is observed in obesity and diabetes.

## Data Availability

Data presented in the current finding are available with the corresponding author and can be accessible upon request.

## Abbreviations

XIST: X-inactive specific transcript
PRC2: Polycomb Repressive complex 2
MALAT1: Metastasis-associated lung adenocarcinoma transcript 1
FENDRR: FOXF1 adjacent non-coding development regulatory RNA
H3K27: Histone H3-lysine 27
HULC: Hepatocellular carcinoma up-regulated long non-coding
UCA1: Urothelial cancer associated 1
PVT1: Plasmacytoma variant translocation 1
NKD2: Naked cuticle homolog 2
EZH2: Enhancer of Zeste Homolog 2
HOTAIR: HOX transcript antisense RNA
DACOR1: DNMT1-associated colon cancer repressed lncrna 1
ANRIL: Antisense non-coding RNA in the INK4 locus
*MEG3*: Maternally expressed 3
GAS5: Growth arrest specific 5
NEAT1: Nuclear paraspeckle assembly transcript 1
TP53TG1: TP53 target 1
GClnc1: Gastric cancer-associated lncRNA 1
SAHA: Suberoylanilide hydroxamic acid
PRKAB2: Protein kinase AMP-activated non-catalytic subunit beta 2
PFKFB3: 6-Phosphofructo-2-kinase/fructose-2,6-biphosphatase 3
SOCS2: Suppressor of cytokine signaling 2
SREBF1: Sterol regulatory element binding transcription factor 1
IRS: Insulin receptor substrate
PPP1R3A: Protein phosphatase 1 regulatory subunit 3A
PPP2R5B: Protein phosphatase 2 regulatory subunit B'Beta
